# Independent Effects of Protein Core Size and Expression on Residue-Level Structure-Evolution Relationships

**DOI:** 10.1371/journal.pone.0046602

**Published:** 2012-10-03

**Authors:** Eric A. Franzosa, Yu Xia

**Affiliations:** 1 Bioinformatics Program, Boston University, Boston, Massachusetts, United States of America; 2 Department of Chemistry, Boston University, Boston, Massachusetts, United States of America; 3 Department of Biomedical Engineering, Boston University, Boston, Massachusetts, United States of America; University of Cyprus, Cyprus

## Abstract

Recently, we demonstrated that yeast protein evolutionary rate at the level of individual amino acid residues scales linearly with degree of solvent accessibility. This residue-level structure-evolution relationship is sensitive to protein core size: surface residues from large-core proteins evolve much faster than those from small-core proteins, while buried residues are equally constrained independent of protein core size. In this work, we investigate the joint effects of protein core size and expression on the residue-level structure-evolution relationship. At the whole-protein level, protein expression is a much more dominant determinant of protein evolutionary rate than protein core size. In contrast, at the residue level, protein core size and expression both have major impacts on protein structure-evolution relationships. In addition, protein core size and expression influence residue-level structure-evolution relationships in qualitatively different ways. Protein core size preferentially affects the non-synonymous substitution rates of surface residues compared to buried residues, and has little influence on synonymous substitution rates. In comparison, protein expression uniformly affects all residues independent of degree of solvent accessibility, and affects both non-synonymous and synonymous substitution rates. Protein core size and expression exert largely independent effects on protein evolution at the residue level, and can combine to produce dramatic changes in the slope of the linear relationship between residue evolutionary rate and solvent accessibility. Our residue-level findings demonstrate that protein core size and expression are both important, yet qualitatively different, determinants of protein evolution. These results underscore the complementary nature of residue-level and whole-protein analysis of protein evolution.

## Introduction

**Figure 1:**
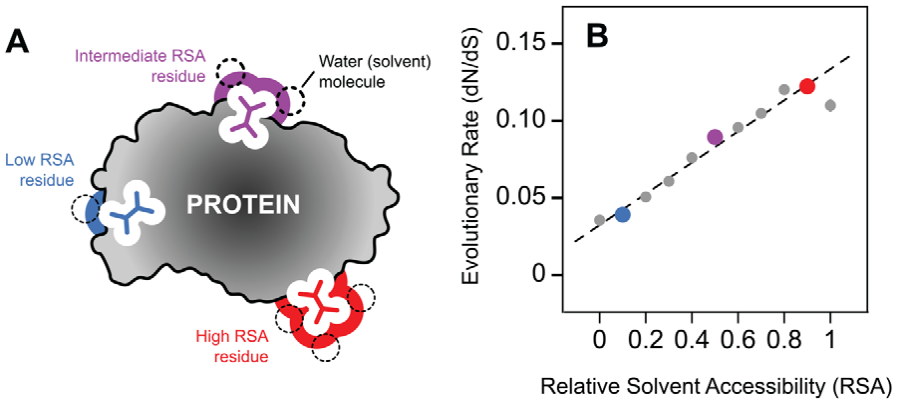
Residue-level structure-evolution relationships. (**A**) A cartoon diagram of a protein shown in cross section, highlighting three residues in different relative solvent accessibility (RSA) microenvironments. (**B**) Evolutionary rate (as measured by dN/dS) scales in a strong, positive, linear manner with RSA, as previously demonstrated [7].

Understanding how protein three-dimensional (3D) structure constrains sequence evolution is an important topic in protein science. Among the most well-known of these structure-evolution relationships is the observation that buried amino acid residues tend to be more conserved in evolution than their solvent-exposed counterparts [1], [2], [3], [4], [5], [6]. Using homology-based 3D structural annotations of yeast proteins, we recently demonstrated that there exists a continuous, linear relationship between residue burial and selective constraint (Figure 1) [7]. This linear relationship has been subsequently confirmed to occur as a result of selection at the amino acid level, and a mechanism has been proposed for the linearity of the trend based on observed site-specific amino acid distributions [8].

In our original work, we demonstrated that the parameters of the linear relationship between a residue's selective constraint (as measured by dN/dS) and burial (as measured by relative solvent accessibility, RSA) were sensitive to protein core size [7]. In yeast proteins with lower average solvent accessibility (“large-core” proteins), we observed that dN/dS increased proportionally faster with increasing solvent accessibility, while the intercept of the trend remained relatively unchanged. Although protein core size has a dramatic effect on structure-evolution relationships at the residue level, its impact on whole-protein evolutionary rate is much smaller, due to the opposing effects of an increased fraction of conserved buried residues and decreased selective constraint on the surface for large-core proteins [7], [9], [10].

In contrast with protein core size, expression level is a dominant determinant of whole-protein evolutionary rate, with highly expressed proteins tending to evolve much more slowly [11], [12], [13]. Some estimates have indicated that protein expression level may explain up to 50% of the variation in protein evolutionary rates within species [14], while basic structural properties (including core size) are proposed to account for only 5 to 10% [9], [10]. Uncovering the mechanisms underlying why highly expressed proteins evolve slowly is therefore a subject of intense investigation. Possible explanations for reduced evolutionary rates among highly expressed proteins include their higher fitness impact [15], selection for translational efficiency [16], selection against misfolding [17], selection against translational error-induced misfolding [14], and selection against mis-interaction [18]. In spite of the widely acknowledged importance of protein expression in determining sequence evolution at the whole-protein level, the impact of protein expression on residue-level structure-evolution relationships remains relatively unexplored.

In this work, we investigate the joint effects of protein core size and expression on the linear relationship between selective constraint and residue burial. We find that, at the residue level, protein core size and expression both have major impacts on protein structure-evolution relationships. However, protein core size and expression influence residue-level structure-evolution relationships in qualitatively different ways. Unlike protein core size, which primarily impacts the evolutionary rate of surface residues but not core residues, increased protein expression level appears to increase selective constraint uniformly throughout the protein, independent of residue solvent accessibility. While protein core size exerts its influence on the residue-level structure-evolution relationship primarily at the level of non-synonymous substitutions, protein expression affects both non-synonymous and synonymous substitution rates. As further support of their mechanistic differences, we go on to demonstrate that protein core size and protein expression act independently to shape the overall selective constraint on amino acid residues.

## Materials and Methods

For this work, we employed a dataset of homology-based 3D structural annotations of yeast (*Saccharomyces cerevisae*) proteins constructed during our previous analysis of residue-level structure-evolution relationships [7]. We assigned yeast proteins to 3D structures in the Protein Data Bank [19] based on sequence homology. Using gapped BLAST [20], we compared each yeast protein to proteins of known 3D structure at the amino acid sequence level, and saved the most significantly aligned structure as a 3D template for the yeast protein if the alignment exhibited strong bidirectional sequence coverage (≥70%). We discarded structures that lacked sufficient atomic detail or that contained large missing regions. This resulted in a dataset of 922 yeast proteins with homology-based 3D structural annotations, based on alignments having average E-value <10^−8^ and average sequence identity of 50%. In the absence of a gap in the alignment, we assigned physical properties calculated for amino acid residues in these structures to the corresponding aligned yeast protein residues. Specifically, we calculated the solvent accessible surface area of amino acid residues using *MSMS* [21] with hydrogen atoms excluded. We normalized raw surface area measurements to the 99^th^ percentile within each residue type to produce *Relative Solvent Accessibility* (RSA), setting outliers to 100% RSA.

Of the 922 yeast proteins with 3D structural models, we were able to pair 795 with their most significantly aligned orthologs in the three closely related yeasts *S. paradoxus*, *S. mikatae*, and *S. bayanus* based on data from the Fungal Orthogroups Repository [22]. These 795 proteins and their homology-based 3D structural annotations constitute the primary dataset for this work, and are listed (along with associated data) in Table S1. In order to divide proteins into large-core and small-core groups, we ranked proteins according to the average RSA of their residues, and designated the bottom third (those with low average RSA) as large-core proteins, and the top third (those with high average RSA) as small-core proteins. In order to divide proteins into low-expression and high-expression groups, we ranked proteins according to their codon adaptation index (CAI) in *S. cerevisiae*, and designated the bottom third (those with low CAI) as low expression proteins, and the top third (those with high CAI) as high expression proteins. CAI is a commonly used sequence-based proxy for expression level that measures enrichment for preferred synonymous codons within a coding sequence [23], [24]. Compared to experimental estimates of protein expression level, CAI has the advantages of being (i) condition-independent and (ii) easily measured for all proteins.

We aligned codons from the four yeast species to their corresponding amino acid positions in the 3D structural models using our protein-level alignments as templates. We then concatenated gapless codon alignments corresponding to amino acids with similar RSA values and analyzed their evolutionary properties using *codeml*, a component of the PAML software package [25]. Specifically, we calculated a single dN/dS ratio for the tree connecting the four closely related yeast species (model  = 0). We estimated the error in our measurements of dN/dS using a bootstrapping procedure. Starting from the original codon alignment for a given RSA bin, we constructed 100 bootstrapped alignments of the same size by randomly sampling aligned codons from the original alignment with replacement and concatenating them. We then recalculated dN/dS for each of these 100 bootstrapped alignments, resulting in a distribution of dN/dS values. The standard deviation of this distribution serves as an estimate of the standard error of the original dN/dS measurement. The same alignments and procedures were used to estimate the codon adaption index of *S. cerevisiae* residues in each bin using *codonw* (http://codonw.sourceforge.net/).

We fit lines to dN/dS versus RSA relationships using a procedure that accounts for variation in the error in dN/dS estimation between RSA bins [26]. For two such lines


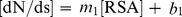
 and 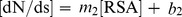


we computed the significance of the difference in their slopes 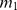 and 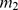 by calculating a *t*-statistic and comparing it to the two-tailed *t*-distribution with 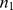
+ 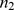 – 4 degrees of freedom, where 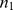 and 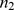 are the numbers of points from the two line fitting procedures. Specifically, we calculated
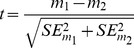
where 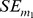 and 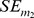 are the standard errors of the slopes of the first and second lines, which are calculated during the line fitting procedure. We used the same procedure to compare the intercepts of the two lines (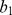 and 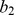). To directly test the significance of the deviation of a slope *m* from the null expectation (*m* = 0), we computed a *t*-statistic as 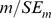
and compared it to the two-tailed *t*-distribution with *n* – 2 degrees of freedom, where *n* is the number of points used in the line fitting procedure. We used the same procedure to test the significance of the deviation of an intercept *b* from the null expectation. In our analyses of fold change in dN/dS between two groups of proteins as a function of RSA, the null expectation for the intercept is *b* = 1 (i.e., no fold change).

Our approach outlined above involves fitting multiple linear models to data separately using a maximum likelihood approach, and then doing further statistical analysis on the estimated parameters (slopes and intercepts). An alternative, one-step approach would be to test our hypotheses directly within the maximum-likelihood framework. The same data can be fitted with either one line (null model) or two lines (alternative model), and a likelihood ratio test can be used to directly compare the fit of the two models. We chose our current two-step approach due to its conceptual simplicity.

Although evolutionary rates and codon bias are calculated from the actual yeast protein sequence, RSA values are directly calculated from the template 3D structure, which may have a different amino acid sequence. Our RSA calculations are based on the assumption that site-specific RSA values are largely dependent on backbone conformation and independent of the identity of the amino acid at the site, and thus well-conserved between close homologs. This assumption is strongly supported by our previous finding [7] that there is a strong correlation (*r* = −0.767) between RSA and the number of Cα atom neighbors, a measure that depends only on backbone conformation and not on side-chain identity. To minimize the impact of any errors introduced by our RSA calculations, we divide residues into relatively broad RSA bins in all of our analyses.

## Results

### Protein core size affects the evolution of surface residues, whereas protein expression affects the evolution of all residues

**Figure 2:**
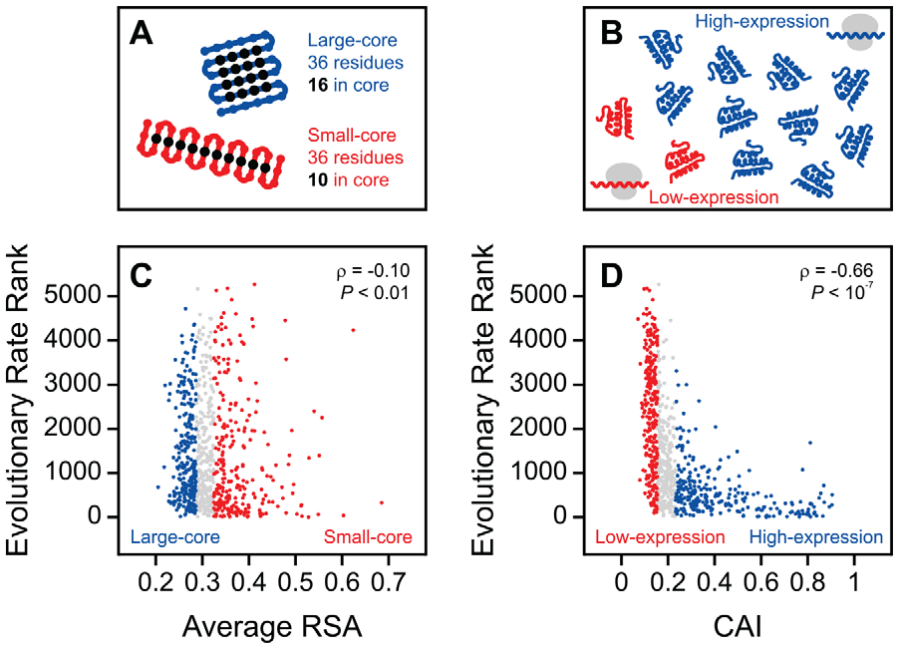
Protein core size, expression level, and evolutionary rate. (**A**) An illustration of protein core size. A large-core protein (blue) contains a greater proportion of buried residues than a small-core protein (red). (**B**) An illustration of protein expression level. (**C**) We divided the 795 proteins in our study into small-core and large-core groups corresponding to the top and bottom third of proteins ranked by average relative solvent accessibility (RSA). Average RSA (core size) is a relatively weak predictor of whole protein evolutionary rate. (**D**) We divided proteins into high-expression and low-expression groups corresponding to the top and bottom third of proteins ranked by codon adaptation index (CAI). CAI is a strong predictor of whole protein evolutionary rate. Rankings of whole-protein evolutionary rate are from [13]; a larger rank implies a faster evolutionary rate (relaxed selective constraint).

In order to evaluate the joint effects of protein core size and protein expression on the residue-level structure-evolution relationship (Figure 1), we annotated 795 yeast proteins and their orthologs in three closely related yeast species with 3D structures based on sequence homology. We then divided these proteins into small-core and large-core groups corresponding to the top and bottom third of proteins ranked by the average relative solvent accessibility (RSA) of their residues (Figure 2 A and C). We divided the same 795 proteins into high-expression and low-expression groups corresponding to the top and bottom third of proteins ranked by codon adaptation index (CAI), a DNA sequence-based proxy for protein expression (Figure 2 B and D). Within each group of proteins, we binned amino acid residues according to RSA and then estimated the degree of selective constraint on residues within each bin (dN/dS). dN/dS compares the rate of non-synonymous amino-acid changing substitutions (dN) to the rate of synonymous substitutions (dS) at the DNA level, with the latter acting as a normalizing factor. At the whole-protein level, core size is a relatively weak predictor of evolutionary rate among these 795 proteins (Figure 2C), while expression level is a strong predictor (Figure 2D), consistent with previous findings [9], [10], [11], [12], [13]. Notably, protein core size and expression level are not significantly correlated within our dataset (Spearman's rank correlation *P* = −0.044, two-tailed *P* = 0.21).

**Figure 3:**
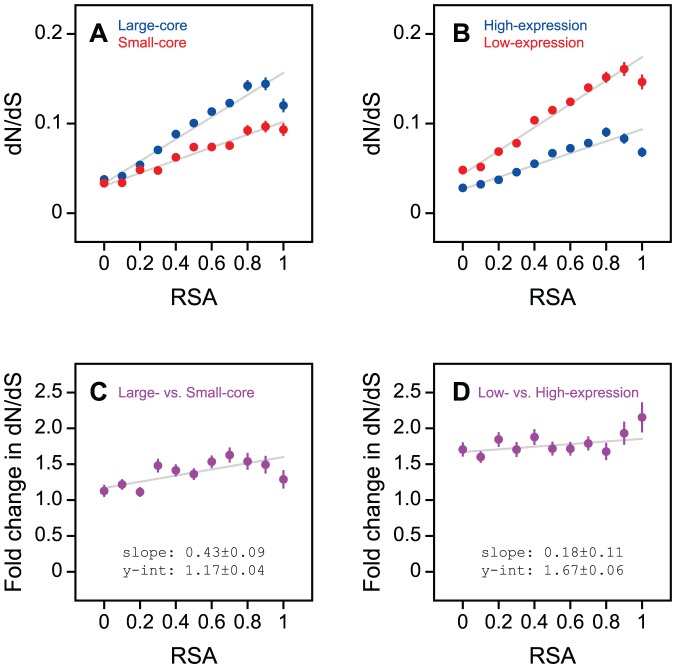
Qualitatively different effects of protein core size and expression level on residue evolution. (**A**) Exposed residues from large-core proteins evolve faster than exposed residues from small-core proteins, while buried residues are similarly constrained. (**B**) Residues from high-expression proteins always evolve more slowly than similarly exposed residues from low-expression proteins. (**C**) Fold change in evolutionary rate varies significantly as a function of solvent accessibility for large-core versus small-core proteins. **(D)** Fold change in evolutionary rate does not vary significantly as a function of solvent accessibility for low-expression versus high-expression proteins. Error measures for slope and intercept reflect the standard error.

As we previously described [7], the difference in dN/dS between residues from large-core versus small-core proteins is very small for completely buried (RSA = 0) residues, but becomes progressively larger for exposed residues (Figure 3A). In contrast, here we show that the difference in dN/dS between residues from low-expression versus high-expression proteins is large for all RSA bins (Figure 3B). This difference is manifested in the best-fit lines relating residue dN/dS to RSA for different groups of proteins: the slope of the dN/dS versus RSA trend for large-core proteins is significantly larger than the slope for small-core proteins (*P*<10^−8^), but the intercepts for the two trends are not significantly different (*P* = 0.11). On the other hand, the slope and intercept of the dN/dS versus RSA trend for low-expression proteins are both significantly larger than the slope and intercept for high-expression proteins (*P*<10^−10^; *P*<10^−8^). Hence, we conclude that the general residue-level structure-evolution relationship (Figure 1B) is sensitive to protein expression, and that the sensitivity to protein expression (Figure 3B) is qualitatively different from the sensitivity to protein core size (Figure 3A).

### Protein expression affects the evolution of all residues uniformly relative to protein core size

Figure 3B would seem to suggest that, because reducing expression level increases the slope of the dN/dS versus relative solvent accessibility (RSA) trend, expression level must affect surface residues more than core residues (i.e., be structure-dependent). However, the following thought experiment reveals that this line of reasoning is incorrect. Imagine that we impose a large, uniform selective constraint over the entirety of a protein's sequence. In this case, we would expect both the intercept and slope of the dN/dS versus RSA trend to approach zero. In other words, the slope of the trend would change in response to a new selective constraint, even though (by definition) the constraint does not depend on structure. Hence, a change in slope is not a good indicator of the structure dependence of a selective constraint. At the same time, although both the slope and intercept of the dN/dS versus RSA trend decrease substantially in this thought experiment, we expect that the *fold change* in dN/dS as a function of RSA would not change. Therefore, we argue that fold change is a much better indicator of the structure dependence of a selective constraint than change in slope alone.

To better understand the nature of the difference between the effects of protein core size and protein expression on residue evolution, we plotted the fold change in dN/dS as a function of RSA for large-core versus small-core proteins (Figure 3C), and for low-expression versus high-expression proteins (Figure 3D). The intercept of the fold change in dN/dS versus RSA trend indicates the difference in selective constraint among completely buried residues with respect to a particular context (protein core size or expression level); an intercept of 1 implies that there is no difference. The slope of the fold change in dN/dS versus RSA trend indicates the degree to which the effects of a given context sensitivity depend on solvent accessibility; a slope of 0 implies that there is no dependence, i.e., the fold change in dN/dS observed for that context is uniform across residues (independent of structure).

In the case of the core size, fold change in dN/dS between large-core and small-core proteins increases significantly with increasing solvent accessibility (*P* = 0.0013), confirming that a residue's solvent accessibility and protein core size interact to dictate the degree of selective constraint on the residue (Figure 3C). In contrast, the large fold change in dN/dS between low-expression and high-expression proteins does not vary significantly with increasing solvent accessibility (*P* = 0.14; Figure 3D). In other words, there appears to be a uniform increase in selective constraint among residues from high-expression proteins that does not depend on structure. Although solvent accessibility and protein expression both contribute to the degree of selective constraint on a residue (Figure 3B), their interaction is weak, especially relative to the significant interaction between solvent accessibility and protein core size.

We performed several controls to ensure that our results were not sensitive to the manner in which we divided proteins into large-core versus small-core and high-expression versus low-expression groups. First, to test that our results were not dependent on our choice of CAI as a measure of protein expression, we divided proteins into high-expression and low-expression groups using two additional estimates of protein expression level: mRNA copies per cell [27] and a meta-analysis of protein abundance experiments [28]. In both cases, repeating the analyses of Figure 3 B and D revealed no significant variation in the fold change in dN/dS between low-expression and high-expression proteins with increasing solvent accessibility, consistent with our original analysis that used CAI as an estimate of protein expression (*P* = 0.65, *P* = 0.39; Figure S1).

As a further control, we repeated the analyses of Figure 3 using different cutoffs to define the large-core versus small-core and high-expression versus low-expression groups. In the original analyses, we designated the top and bottom third of proteins ranked by increasing average residue RSA as small-core and large-core, and the top and bottom third of proteins ranked by increasing CAI as high-expression and low-expression (Figure 2 C and D). Using the same rankings, we repeated our analyses first by designating the top and bottom 50% of proteins as the extremes of core size and protein expression (Figure S2), and again by designating the top and bottom 25% of proteins as the extremes of core size and protein expression (Figure S3). In each case, the results remained consistent with our original findings: fold change in dN/dS between large-core and small-core proteins increased significantly with increasing solvent accessibility (*P* = 0.012, *P* = 0.0035), while fold change in dN/dS between high-expression and low-expression proteins did not (*P* = 0.38, *P* = 0.57; Figures S2 and S3).

### The different effects of protein core size and expression on residue evolution are driven by selection at the amino acid level

**Figure 4:**
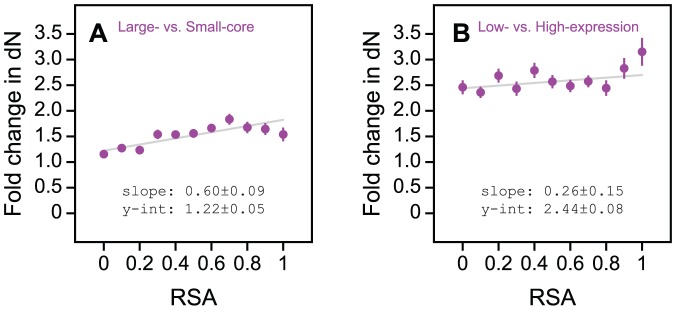
Fold change in selection at the amino acid sequence level as a function of solvent accessibility across protein core size and expression contexts. (A) Fold change in the rate of amino acid sequence evolution (dN) varies significantly as a function of solvent accessibility for large-core versus small-core proteins. (B) Fold change in dN does not vary significantly as a function of solvent accessibility for low-expression versus high-expression proteins.

The dN/dS ratio is based on the assumption that synonymous DNA mutations are selectively neutral, and hence their rate of fixation (dS) can be used as a normalizing factor for comparing the rates of fixation of amino acid changing mutations (dN) between protein coding sequences. This assumption must be critically considered in evolutionary analyses involving highly expressed proteins, which tend to be encoded by preferred synonymous codons (i.e., these proteins have experienced selection at the level of synonymous mutations). To test the dependence of our results on selection for synonymous codons, we repeated our previous analyses using un-normalized dN as a proxy for selective constraint (Figure 4). Results based on fold change in dN are consistent with our previous conclusions: for large-core versus small-core proteins, fold change in dN increases significantly with increasing solvent accessibility (*P* = 0.00011; Figure 4A), but not for low-expression versus high-expression proteins (*P* = 0.11; Figure 4B). Hence, our conclusions about how the protein core size and expression level contexts interact with solvent accessibility to determine residue-level selective constraint are not confounded by selection at the level of synonymous codons.

**Figure 5:**
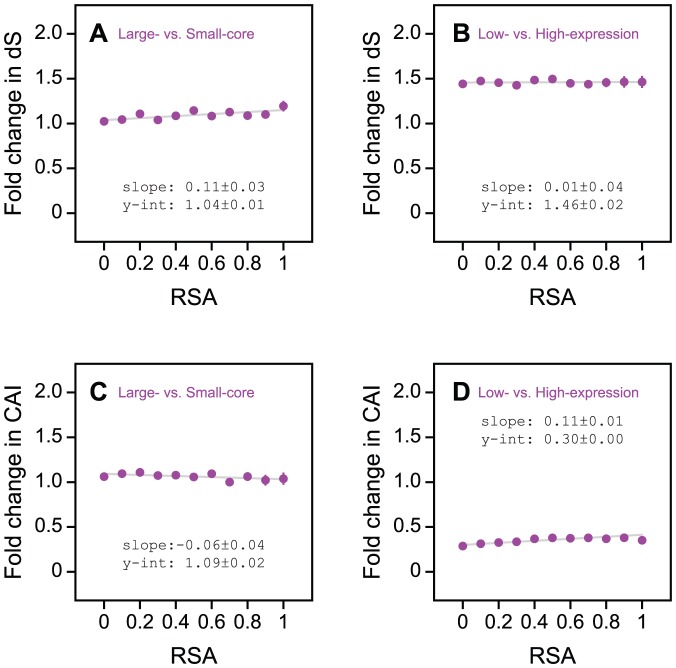
Fold change in selection at the synonymous codon level as a function of solvent accessibility across protein core size and expression contexts. (A) Large-core versus small-core proteins experience minimal difference in the degree of synonymous codon selection that they experience. (B) Highly expressed proteins experience a uniform increase in selection at the level of synonymous codons throughout their coding sequences. (C) Large-core versus small-core proteins experience minimal difference in their use of preferred codons (as measured by codon adaptation index, CAI). (D) Highly expressed proteins experience a relatively uniform increase in their use of preferred codons.

For large-core versus small-core proteins, fold change in dS deviates only marginally from 1 with increasing relative solvent accessibility (RSA), suggesting that there is relatively little difference in the degree of synonymous selection acting on these two groups of proteins (Figure 5A). On the other hand, Figure 5B shows that for low-expression versus high-expression proteins, fold change in dS is much larger than 1 for buried residues (*P*<10^−9^), and does not change significantly with increasing solvent accessibility (*P* = 0.89). This implies that highly expressed proteins experience a uniform (structure independent) increase in selective constraint at the level of synonymous codons.

To further explore the effects of synonymous selection on residue-level structure-evolution relationships, we calculated the fold change in codon adaption index (CAI) as a function of solvent accessibility over the core size and protein expression contexts (Figure 5 C and D). The results were largely consistent with our findings based on fold change in dS. For large-core versus small-core proteins, we observe minimal variation in fold enrichment for preferred codons (CAI) across sites of different RSA values (Figure 5C). For low-expression versus high-expression proteins (Figure 5D), we observe significant variation in CAI fold change with increasing RSA (*P*<10^−5^), but the trend is relatively weak and does not seem to affect rates of synonymous evolution across these proteins (Figure 5B).

### Independent effects of protein core size and expression on residue evolution

**Figure 6:**
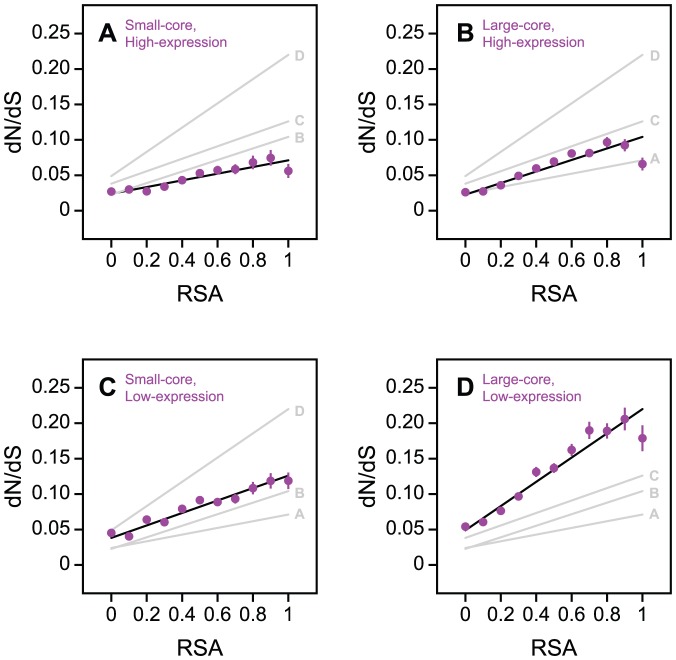
Independent effects of protein core size and expression on residue evolution. The dN/dS versus solvent accessibility relationship for four different protein groups: (**A**) small-core and high-expression, (**B**) large-core and high-expression, (**C**) small-core and low-expression, and (**D**) large-core and low-expression. Increasing core size and decreasing expression level simultaneously in (D) results in significant increases to the slope of the trend relative to either separate change. The best-fit lines for all four groups of proteins are replicated in each panel for comparison.

Finally, to evaluate the degree of independence between protein core size and protein expression in shaping protein evolution at the residue level, we divided proteins into four non-overlapping groups: (i) small-core and high-expression, (ii) large-core and high-expression, (iii) small-core and low-expression, and (iv) large-core and low-expression. We then repeated our procedure of binning residues according to relative solvent accessibility (RSA) and calculating dN/dS for each group of proteins (Figure 6). Residues from high-expression, small-core proteins tend to be the most conserved, but still experience a significant increase in dN/dS with increasing RSA (*P*<10^−4^; Figure 6A). Increasing core size or decreasing expression level separately both result in an increase in the slope of the dN/dS versus RSA trend (Figure 6 B and C). Moreover, increasing core size and decreasing expression level simultaneously (Figure 6D) results in a significant increase in the slope of the trend relative to either separate change (*P*<10^−7^ in both comparisons), confirming that protein core size and protein expression contribute separately to the residue-level structure-evolution relationship.

## Discussion

Relative solvent accessibility, a structural property, is an important determinant of evolution at the level of individual amino acid residues. However, at the level of whole proteins, expression level appears to be the most dominant force influencing average evolutionary rate. In this work, we sought to understand the interplay between these two phenomena.

A protein's overall core size is not a dominant predictor of its average evolutionary rate. However, the protein core size “context” interacts with a residue's microenvironment in an important way to dictate selective constraint. While completely buried residues appear to be highly conserved in all proteins, surface residues evolve proportionally faster in large-core proteins. This results in variation in the fold change in selective constraint between small-core and large-core proteins as solvent accessibility increases. In comparison, we find that protein expression appears to affect selective constraint uniformly throughout a protein. Although residues from highly expressed proteins tend to be slow evolving, there is no significant variation in the fold change in selective constraint between high-expression and low-expression proteins with increasing solvent accessibility. Hence, we conclude that compared to protein core size, although protein expression is an important determinant of average evolutionary rate, it appears to act in a manner that is largely independent of protein structure.

The mechanisms by which protein core size and expression level influence structure-evolution relationships at the residue level are qualitatively different, and combine to produce large effects. In particular, we find that increasing core size and decreasing expression level both increase the slope of the evolutionary rate versus solvent accessibility relationship. As a result, surface residues from large-core, lowly expressed proteins are among the fastest evolving, and evolve significantly faster than surface residues from generic large-core proteins as well as generic low-expression proteins.

Our work indicates that, compared to changing protein core size, raising the expression level of a protein increases selective constraint relatively uniformly throughout the coding sequence. This observation has important implications for our mechanistic understanding of the anti-correlation between protein expression and evolutionary rate at the whole protein-level. The observation that the effects of protein expression on residue evolution are largely independent of structure is consistent with structure-independent mechanisms, e.g. selection for translational efficiency [16]. This mechanism would be consistent with the observed uniform fold increase in selection at the amino acid level (e.g., favoring metabolically “cheap” amino acids throughout the protein), and with the observed uniform fold increase in selection at the level of synonymous codons (e.g., favoring abundant tRNA species).

Recently, several structure-dependent mechanisms have been proposed suggesting that heightened selection on highly expressed proteins acts (i) to minimize misfolding by directly stabilizing the native structure [17], (ii) to minimize misfolding by enhancing robustness against translational errors [14], or (iii) to minimize mis-interaction [18]. It is expected that all of these mechanisms contribute to the whole protein-level expression-evolutionary rate anti-correlation, but the net effects on core and surface residues must balance to produce the observed relatively uniform fold decrease in evolutionary rate throughout the entire sequence of highly expressed proteins. Our results put an upper limit on the degree of structure dependence of the overall effects of protein expression on residue evolution.

Our results further highlight the power of residue-level analysis of protein evolution as compared to whole protein-level analysis. At the whole protein level, protein expression is a much more dominant determinant of protein evolution than core size. In contrast, at the residue level, protein expression and core size are both dominant determinants of protein evolution. In addition, protein expression and core size affect residue evolution in qualitatively different ways. All of these patterns are hidden in whole protein-level analysis, and can only be revealed by residue-level analysis. These results underscore the importance of residue-level structural analysis of protein evolution.

**Note.** After submission of this manuscript, we were made aware of a related study [29] on the effects of protein core size and expression level on the linear relationship between residue-level dN/dS and solvent accessibility. That study provides further evidence that while protein core size affects only the slope, expression level affects both the slope and the intercept.

## Supporting Information

Figure S1 **Alternative measures of protein expression yield similar dN/dS versus RSA relationships for low-expression versus high-expression proteins.** This is a control study of Figure 3 B and D from the main text using two different experimental estimates of protein expression, mRNA abundance and protein abundance. **(A)** Residues from proteins with high mRNA abundance always evolve more slowly than similarly exposed residues from proteins with low mRNA abundance. **(B)** Residues from proteins with high protein abundance always evolve more slowly than similarly exposed residues from proteins with low protein abundance. **(C)** Fold change in evolutionary rate does not vary significantly as a function of solvent accessibility between low versus high mRNA abundance proteins (*P* = 0.65). **(D)** Fold change in evolutionary rate does not vary significantly as a function of solvent accessibility between low versus high protein abundance proteins (*P* = 0.39).
(EPS)

Figure S2 **Qualitatively different effects of protein core size and expression on residue evolution (alternate grouping strategy I).** This is a control study of Figure 3 from the main text in which the “extreme” values of protein core size and expression level are defined by the top and bottom 50% of proteins ranked by average RSA and CAI, respectively. **(A)** Exposed residues from large-core proteins evolve faster than exposed residues from small-core proteins, while buried residues are similarly constrained. **(B)** Residues from high-expression proteins always evolve more slowly than similarly exposed residues from low-expression proteins. **(C)** Fold change in evolutionary rate varies significantly as a function of solvent accessibility between large-core versus small-core proteins (*P* = 0.012). **(D)** Fold change in evolutionary rate does not vary significantly as a function of solvent accessibility between low-expression versus high-expression proteins (*P* = 0.39).
(EPS)

Figure S3 **Qualitatively different effects of protein core size and expression on residue evolution (alternate grouping strategy II).** This is a control study of Figure 3 from the main text in which the “extreme” values of protein core size and expression level are defined by the top and bottom 25% of proteins ranked by average RSA and CAI, respectively. **(A)** Exposed residues from large-core proteins evolve faster than exposed residues from small-core proteins, while buried residues are similarly constrained. **(B)** Residues from high-expression proteins always evolve more slowly than similarly exposed residues from low-expression proteins. **(C)** Fold change in evolutionary rate varies significantly as a function of solvent accessibility between large-core versus small-core proteins (*P* = 0.0035). **(D)** Fold change in evolutionary rate does not vary significantly as a function of solvent accessibility between low-expression versus high-expression proteins (*P* = 0.57).
(EPS)

Table S1 **Details of the 795 yeast proteins and their structural models used in this study.** Columns list yeast protein (ORF), structural model (PDB structure ID underscore chain ID), BLAST-based alignment statistics, average relative solvent accessibility (core size) bin, and protein expression bin.
(XLSX)
